# Patterns of mRNA and protein expression during minus-lens compensation and recovery in tree shrew sclera

**Published:** 2011-04-12

**Authors:** Hong Gao, Michael R. Frost, John T. Siegwart, Thomas T. Norton

**Affiliations:** Department of Vision Sciences, University of Alabama at Birmingham, Birmingham, AL

## Abstract

**Purpose:**

To increase our understanding of the mechanisms that remodel the sclera during the development of lens-induced myopia, when the sclera responds to putative “go” signals of retinal origin, and during recovery from lens-induced myopia, when the sclera responds to retinally-derived “stop” signals.

**Methods:**

Seven groups of tree shrews were used to examine mRNA levels during minus lens compensation and recovery. Starting 24 days after eye opening (days of visual experience [VE]) lens compensation animals wore a monocular –5D lens for 1, 4, or 11 days. Recovery animals wore the –5D lens for 11 days, which was then removed for 1 or 4 days. Normal animals were examined at 24 and 38 days of VE. All groups contained 8 animals. Scleral mRNA levels were examined in the treated and contralateral control eyes with quantitative real-time polymerase chain reaction (qPCR) for 27 genes divided into four categories: 1) signaling molecules, 2) matricellular proteins, 3) metalloproteinases (MPs) and tissue inhibitors of metalloproteinases (TIMPs), and 4) cell adhesion and other proteins. Four groups (n=5 per group) were used to examine protein levels. One group wore a –5D lens for 4 days. A second group recovered for 4 days after 11 days of −5D lens treatment. Two groups were used to examine age-matched normal protein levels at 28 and 39 days of VE. The levels of six scleral proteins that showed differential mRNA expression were examined with quantitative western blots.

**Results:**

Nineteen of the genes showed differential (treated eye versus control eye) expression of mRNA levels in at least one group of animals. Which genes showed differential expression differed after 1 and 4 days of compensation and after 1 or 4 days of recovery. The mRNA level for one gene, a disintegrin and metalloproteinase with thrombospondin motifs 1 (*ADAMTS1*), was upregulated in the treated eyes after 1 day of compensation. After 4 days, transforming growth factor beta receptor 3 (*TGFBR3*), transforming growth factor-beta-induced protein ig-h3 (*TGFBI*), and matrix metalloproteinase 14 (*MMP14*) mRNA levels were upregulated. Downregulated were mRNA levels for transforming growth factor beta-1 (*TGFB1*), transforming growth factor beta-2 (*TGFB2*), thrombospondin 1 (*THBS1*), tenascin (*TNC*), osteonectin (*SPARC*), osteopontin (*SPP1*), tissue inhibitor of metalloproteinases 3 (*TIMP3*), and a disintegrin and metalloproteinase with thrombospondin motifs 5 (*ADAMTS5*). After 11 days of lens wear, there was no differential expression. During recovery, after 1 day, treated-eye mRNA downregulation was found for *TGFB2*, *TGFBR1*, *TGFBR2*, *TGFBR3*, *SPARC*, *ADAMTS1*, *ADAMTS5*, syndecan 4 (*SDC4*), and collagen type VI, alpha 1 (*COL6A1*). After 4 days, *TGFB1*, *TGFB2*, *TGFB3*, *THBS2*, and *TIMP3* mRNA levels were upregulated in the recovering eye. Significant downregulation, relative to normal eyes, was found in both the control and treated eyes for most genes after 1 day of compensation; a similar decrease was found, compared to lens-compensated eyes, after one day of recovery. Protein levels for THBS1 showed positive correlation with the differential mRNA levels and TGFBR3 showed a negative correlation. No differential protein expression was found for TGFB2, TGFBI, MMP14, and TIMP3.

**Conclusions:**

The different patterns of differential mRNA expression during minus lens compensation (hyperopia) and recovery (myopia) show that scleral fibroblasts distinguish between “go” and “stop” conditions. There is evidence of binocular global downregulation of genes at the start of both lens wear and recovery. As additional information accumulates about changes in gene expression that occur during compensation and recovery the “signature” of differential changes may help us to understand in more detail how the sclera responds in “go” and “stop” conditions.

## Introduction

Refractive errors occur when there is a mismatch between the axial length of an eye and its refractive power, which is produced by the cornea, lens and anterior chamber depth. When the axial length matches the optical power, without accommodation, the images of distant objects are focused sharply on the photoreceptors and the eye is emmetropic. However, if the eye’s axial length becomes longer than the focal plane, the images are focused in front of the retina and the eye is myopic. Juvenile-onset myopia affects a significant fraction of the world’s population [1].

As shown by studies in animal models (fish, chicks, monkeys, guinea pigs, tree shrews, and other species) [2-6] and humans [7], an emmetropization mechanism uses visual signals during the early postnatal period to guide the axial elongation rate (primarily the vitreous chamber depth) so that the retina comes to be located close to the focal plane, typically producing eyes that are slightly hyperopic. The emmetropization mechanism can be stimulated with minus powered lenses to produce accelerated axial growth.

A minus-power lens shifts the focal plane posteriorly, moving it behind the retina and making the lens-wearing emmetropic eye hyperopic. In juvenile tree shrews, small mammals (~150 g), closely related to primates [8] with a well characterized emmetropization mechanism [9,10], this stimulates the emmetropization mechanism to produce a compensatory increase in the axial elongation rate above the normal baseline rate and leads to the eye becoming emmetropic while wearing the lens. Nearly all the increased elongation is due to an increase in vitreous chamber depth; there is little change to the cornea or lens [11-14]. Refractive and axial changes can be detected as soon as 2 days after the start of monocular −5 diopter (D) lens wear [9]; after 11 days the compensation is typically complete [9] so that the refractive state, measured with the lens in place, is equal to that of the untreated control eye or age-matched normal eyes.

When the lens is removed after minus lens compensation, the eye initially is myopic. Over time, the refractive state of the eye generally recovers until the refractive power of the eye is again the same as the control and age-matched normal eyes [9,11,15]. Axial length (vitreous chamber) measures have shown that the recovery occurs primarily because there is slowing of the axial elongation rate below normal, while the eye’s optical power continues to mature [16,17]. When recovery is complete, the axial length in the recovered eye matches that of normal and untreated control eyes.

It is generally thought that this visually-guided feedback loop begins with retinally-generated “go” signals that eventually produce an increased axial elongation rate during compensation [18,19]. During recovery, retinally-generated “stop” signals produce a slowed elongation rate [18,20]. The axial elongation rate of the eye is primarily determined by the rate of expansion of the scleral shell, which is a dense extracellular matrix (ECM) connective tissue produced by fibroblast cells [21-23]. In tree shrews, it is comprised largely of collagen fibrils (approximately 90% of scleral dry weight), predominantly fibrillar type I collagen [24,25]. These are arranged in layers (lamellae), along with elastin, proteoglycans, glycoproteins, hyaluronan, and other proteins.

The mechanical properties of the sclera change during minus lens compensation and recovery [11]. During compensation for a –5D lens, scleral creep rate, a measure of viscoelasticity, initially rises with a peak after 4 days of lens wear and then gradually declines as the eye completes its compensation. The creep rate changes occur in parallel with the rise and decline of the axial elongation rate. This change in the sclera may allow normal intraocular pressure to expand the globe during compensation, perhaps by increasing the ease with which the scleral lamellae slip across each other. During recovery, the creep rate rapidly (< 24 h after lens removal) returns to normal levels, and in some cases, drops below normal levels (Siegwart, unpublished data, 2007). Although it appears that “go” and “stop” signals of retinal origin produce changes in the biochemical composition of the sclera which, in turn, control its viscoelasticity, it is unclear which of the scleral structural components, signaling molecules, enzymes and their inhibitors, cell adhesion molecules, and other substances are changed during scleral remodeling.

Previous studies suggest that the process that occurs in the all-fibrous tree shrew sclera during lens compensation and recovery is tissue remodeling rather than modulation of growth. During minus lens compensation, there is a reduction in scleral dry weight (~4%) of the treated eyes due to a net loss of extracellular matrix (ECM) proteins, hyaluronan, and other glycosaminoglycans (GAGs) [17]. During recovery from induced myopia, there is little rebound of dry weight [17] or type I collagen levels [25], although mRNA levels for type I collagen and other genes that changed during myopia development have generally been found to either reverse in direction or to return to normal. From these studies a general picture has emerged of increased turnover and loss of ECM during myopia development and a partial reversal during recovery. Numerous genes are involved in tissue remodeling and studies in tree shrews have found modulation of scleral mRNA levels after minus lens wear or form deprivation and, to a more limited extent, during recovery [16,26-30]. The regulation of mRNA levels is selective, so that levels for some substances, but not all, differ in the treated eyes, relative to control eyes, during either minus lens compensation or recovery [16]. There is evidence that the transforming growth factor beta (TGFβ) signaling pathway plays a major role in the initiation and control of the remodeling process [31].

Despite previous studies, only a relatively small number of the potential candidate genes have been examined. In this study, 27 genes representing four functional groups were selected for examination at the mRNA level by quantitative real-time polymerase chain reaction (qPCR) during lens compensation and recovery: 1) signaling molecules, 2) matricellular proteins, 3) metalloproteinases [MPs] and tissue inhibitors of metalloproteinases [TIMPs], and 4) cell adhesion and other proteins. The 27 genes chosen for this study are meant to be representative of the tissue remodeling process. They are either known to be involved in tissue remodeling in general or have been specifically implicated in scleral remodeling. A subset of six candidates whose mRNAs were found to be differentially expressed was examined at the protein level using quantitative western blots. We hypothesized that, during compensation, specific molecules initiate and participate in ECM remodeling, and during recovery some of the same molecules produce a reversed pattern of mRNA expression, while other molecules are activated or return to normal. The expression signature of the changes may help us to understand how the sclera responds to “go” and “stop” signals received from the retina.

## Methods

### Experimental groups

Juvenile tree shrews (*Tupaia glis belangeri*) were raised by their mothers in our breeding colony on a 14 h light/10 h dark cycle. All procedures complied with the ARVO Statement for the Use of Animals in Ophthalmic and Visual Research and were approved by the Institutional Animal Care and Use Committee of the University of Alabama at Birmingham. Experimental groups were balanced to include both males and females, and avoided pups from the same parents wherever possible. The treated eye was selected to include both right and left eyes and was approximately balanced within and across groups.

#### Gene expression study

Three groups of minus lens compensation animals (n=8 per group) wore a monocular –5D (spherical power) lens starting at 24±1 days after natural eye opening (days of visual experience [VE]), with lens wear continuing for 1, 4, or 11 days. The untreated fellow eye served as a control. Two recovery groups (n=8) wore the monocular –5D lens for 11 days, also starting at 24 days of VE, typically resulting in full compensation. Lenses were removed and the now-myopic treated eye was allowed to recover for either 1 or 4 days. Two ‘age-matched’ normal groups (n=8) were also used – one with 24 days of VE for comparison with the 1 and 4 day compensation groups, and one with 38 days of VE for comparison with the 11 day compensation, 1 day recovery, and 4 day recovery groups.

#### Protein expression study

Four groups (n=5 per group) were used to examine protein levels. One group wore a –5D lens for 4 days. A second group recovered for 4 days after 11 days of −5D lens treatment. Two groups were used to examine age-matched normal protein levels at 28 and 39 days of VE. The levels of six scleral proteins (transforming growth factor beta-2 [TGFB2], transforming growth factor beta receptor 3 [TGFBR3], transforming growth factor-beta-induced protein ig-h3 [TGFBI], matrix metalloproteinase 14 [MMP14], and tissue inhibitor of metalloproteinases 3 [TIMP3]) that showed differential mRNA expression were examined with quantitative western blots.

### Lens wear

To attach the goggle containing the –5D lens firmly to the head during lens treatment, animals were anesthetized (17.5 mg ketamine, 1.2 mg xylazine; supplemented with 0.5−2.0% isoflurane as needed) and received a dental acrylic pedestal at 21±1 days of VE, following procedures described by Siegwart and Norton [32]. Three days later, the goggle frame with a monocular –5D lens (PMMA contact lens, 12 mm diameter; Conforma Contact Lens, Norfolk, VA) was clipped to the pedestal, holding the lens in front of the treated eye. The control eye had unrestricted vision through an open (no lens) goggle frame. The normal groups did not wear a goggle. The goggle was removed for approximately 2 min in dim illumination twice daily (approximately 9:30 AM and 4:30 PM) while the lens was cleaned. During lens cleaning, the animals were kept in a darkened nest box to minimize exposure to visual stimuli. Badly scratched lenses were replaced as needed while the animal was kept in darkness (<30 min).

### Axial and refractive measures

At the time the pedestal was attached, ocular component dimensions were measured under anesthesia with A-scan ultrasound as described by Norton and McBrien [33]. Terminal A-scan measures were omitted out of concern that protracted anesthesia might interfere with retino-scleral signaling. At the start and end of the compensation and recovery periods, non-cycloplegic measures of refractive state were taken on the animals while they were awake using a Nidek ARK 700-A infrared autorefractor (Marco Ophthalmic, Inc., Jacksonville, FL) [34]. Measures were taken with the –5D lens in place and with it removed to measure both the amount of lens compensation and the amount of induced myopia. Cycloplegic refractive measures were omitted to prevent any possible effect on retino-scleral signaling by atropine. Previous studies have found that non-cycloplegic awake autorefractor measures provide a valid estimate of the amount of induced myopia in tree shrews. Actual values for each eye differ from the cycloplegic measures by less than 1 D, and the treated-eye versus control-eye differences are nearly identical between non-cycloplegic and cycloplegic measures [35].

### Tissue collection

On completion of the final refractive measures, animals were anesthetized (17.5 mg ketamine, 1.2 mg xylazine) and then received a lethal dose of sodium pentobarbital (approximately 333 mg/kg) or xylazine (approximately 375 mg/kg). With the animals under deep anesthesia, both eyes were enucleated and placed into either RNAlater solution (Ambion, Austin, TX) for the gene expression groups or chilled dissection buffer (250 mM sucrose, 10 mM Tris, pH 7.0) for the protein expression groups. Extraocular muscles, conjunctiva, and orbital fat were trimmed from the exterior surface of the eye and the cornea cut away. After removing the lens and vitreous humor, the inner and outer scleral surfaces were gently scraped to remove the retina, RPE, choroid, and any remaining extraocular tissue, before freezing the tissue in liquid nitrogen.

### Gene expression analysis

Frozen sclera was pulverized to a fine powder in a chilled Teflon freezer mill (B. Braun Biotech, Allentown, PA) from which total RNA was isolated using a RiboPure kit (Ambion) according to the manufacturer’s instructions, with the addition of an on-filter DNase treatment. The purified RNA was quantified using a NanoDrop spectrophotometer (NanoDrop Technologies, Rockland, ME) with an average yield per sclera of 6.8±1.9 µg (mean±SD). RNA quality was confirmed by denaturing gel electrophoresis (RNA FlashGel; Lonza, Wilmington, DE). cDNA was synthesized from 1 µg of total RNA in a total reaction volume of 20 µl using a Superscript III RT kit (Invitrogen, Carlsbad, CA) with minor modifications (2.5 µM anchored oligo (dT)_20_ primers and DTT omitted). The reaction was terminated by heating at 95 °C for 10 min and the cDNA then diluted fivefold and stored at –20 °C until use. To minimize potential variation, all 8 animals in a given treatment group were processed (RNA extraction and reverse transcription) at the same time.

Tree shrew-specific primers for SYBR Green assays were designed for 27 target genes and the reference gene RNA polymerase II (*POLR2A*) using Beacon Designer 7 (Premier Biosoft  International, Palo Alto, CA; Table 1). Initially, for the majority of candidates, human-specific primers were developed in regions that showed cross-species homology. The PCR product generated by these primers from tree shrew cDNA was then sequenced to allow the design of tree shrew-specific qPCR primers. For some candidates (apolipoprotein A-I [*APOA1*], apolipoprotein E [*APOE*], syndecan 4 [*SDC4*], and transforming growth factor beta receptor 1 [*TGFBR1*]) tree shrew cDNA sequence was available directly. All primers were designed to work under the same cycling conditions. All resulting amplicons were located within the coding region and most spanned at least one intron; amplicon identity was verified by gel electrophoresis and sequencing.

**Table 1:** Primers used to examine gene expression using qPCR.

** **	**Primer sequence**		** **	** **	** **
**Gene**	**Forward**	**Reverse**	**Size**	**Spans intron**	**Efficiency**
**Signaling pathways**					
*TGFB1*	ACCAGAAATACAGCAACAATTCC	AACCCGTTGATGTCCACTTG	205	Yes	98
*TGFB2*	GCAGAGTTTAGGGTCTTTCGTTTG	CTCGTGAACAGCATCAGTTACATC	189	Yes	104
*TGFB3*	ATCACCATAACCCGCATCTAATCC	CGCACACAGCAGTTCTCCTC	139	Yes	91
*TGFBR1*	GACCTCCCAACTACTGTAAAGC	ATCCTCTTCATTTGGCACTCG	162	Yes	89
*TGFBR2*	GCTGCCTGTGTGACTTTGG	TCCTGGATTCTAGCACTTCTGG	123	Yes	94
*TGFBR3*	CCCTGGTCTGGCGTCTGAAG	GTAACTGCTCCATACTCGTTTCGG	190	Yes	87
*FGF2*	GGGTCGTGTCTATCAAAGGAG	ACATTTAGAAGCCAGCAGTCG	80	Yes	90
*FGFR1*	CCTGGAGGTCATCATCTACTGC	AGAGTTCATGGAAGCACTGGAG	196	Yes	96
*APOE*	GGTGCAGACGCTGTCTGACCA	CCTCCAACTCCGCCTTGTAGG	122	Yes	89
*APOA1*	GCTGTGGTATTGACCTTGGCTGT	TTGGCTAAATCCCGCACTCG	110	Yes	100
**Matricellular proteins**					
*THBS1*	CTGTCAGAACTCAGTCACCATC	CCACGGAGACCAGCCATC	136	Yes	81
*THBS2*	GAGACCGACTTCAGGAACTTC	CGAAACCCACTGCGATGC	142	Yes	88
*TNC*	AGACGCCAAGACTCGCTACAG	CAGGTTGACACGGTGACAGTTC	184	Yes	88
*SPARC*	GCGAGTTTGAGAAGGTGTGC	GCCCGATGTAGTCCAGGTG	126	Yes	78
*SPP1*	CCGACGACACCGACCATCC	GGCTTTGACCTCACTCTGTAAACC	190	No	83
**MP/TIMP**					
*MMP3*	GCCATCCGAGGAAATGAGG	TGTCTCTTCTCGTCAAATCTCC	164	Yes	95
*MMP14*	CCCTGGAACCTGGCTACCC	ATAGGTCTTTCCATTGGGCATCC	104	No	104
*ADAMTS1*	TGGCAAAAGCAGCACAACCC	CACAGGTCTGAGCCCCACAC	100	Yes	92
*ADAMTS5*	TCTTCCATCCTAACCAGCATTG	GGTGGCATCATAAGTCTGTCC	165	Yes	105
*TIMP3*	CCGTGTCTATGATGGCAAGATG	ACAAAGCAAGGCAGGTAATAGC	153	Yes	82
**Cell adhesion/other**					
*SDC2*	TGATGACGACTACGCTTCTGC	CAGGCATCTTGTTCTGTGTCTTC	155	Yes	90
*SDC4*	CAAGGAACTGGAGGAGAATG	GGAAAGTGGCAAAGAGGAG	182	Yes	80
*VIM*	GCTCACCAACGACAAGGC	CAGAGTGCTTTCGGCTTCC	121	Yes	94
*COL6A1*	CGACATCCTGTTCGTGCTGG	ATCTGGTTGTGGCTGTACTGC	168	Yes	78
*FN1*	GCAACTCATCAGCATCCAGCAATATG	GGAAACCCAGGAGACCACAAAGC	190	Yes	84
*PEDF*	CCTGAAAGCAACCCAGAACTTGA	GACTTGGTAACTTCGCCTTCGTAAC	147	Yes	90
*TGFBI*	CCTCGGCACTCATCTCTCC	GCAAATTCTTCATCTTGGCATCG	107	Yes	96
**Reference**						
*POLR2A*	CTACCAGCCCCAAGTATTC	GGTGAGTAAGTAGGAGACG	106	No	101

Relative gene expression was quantified on an iQ5 real time PCR system using iQ SYBR Green Supermix (Bio-Rad, Hercules, CA). Reactions were performed in triplicate for all but 90 of the 1,512 total assays (94%); the remaining were performed in duplicate. Random deletion of one of the replicates from the triplicate runs did not change the statistical significance of the results which suggests that the duplicate reactions are valid measures. For each target gene reactions were performed in a 15 µl volume containing 300 nM each primer and the equivalent of 0.5 µl cDNA template. The same cycling parameters were used for all primer sets: 95 °C ×3 min followed by 40 cycles of 95 °C ×15 s, 62 °C ×40 s. Single gene products were obtained for all reactions as assessed by melt curve analysis or gel electrophoresis. The ∆∆Ct method was used [36], first to normalize the expression of the target gene to the reference gene, and then to compare the relative expression of the target gene between treated and control eyes, treated and normal eyes, control and normal eyes. The geometric group mean (for the 8 biological replicates) of these expression ratios was used to calculate the fold change in gene expression for each of the target genes. Paired *t*-tests were used to assess treated versus control eye differences. Unpaired *t*-tests, assuming equal variance, were used to test for differences between all independent groups. The SEM for unpaired comparisons was calculated using a pooled variance. For all comparisons p<0.05 was considered significant.

### Protein expression analysis

The relative expression levels of 6 proteins, whose mRNA levels were found to change in the gene expression analysis, were examined by fluorescent western blotting: TGFB2, TGFBR3, TGFBI, MMP14, and TIMP3; glyceraldehyde-3-phosphate dehydrogenase (GAPDH) was used as a reference protein. Frozen sclera was pulverized to a fine powder in a chilled Teflon freezer mill (B. Braun Biotech, Allentown, PA) and then suspended in 400 µl RIPA extraction buffer (50 mM tris base, 150 mM NaCl, 1% IGEPAL CA-630, 0.5% sodium deoxycholate, 0.1% SDS, protease inhibitor cocktail [# P8340; Sigma, St. Louis, MO], pH 8.0). After incubation on ice for 60 min, the homogenate was centrifuged at 21,000× g for 20 min at 4 °C to pellet cellular debris; the supernatant was collected and its protein content quantified (2D quant kit; GE Healthcare, Piscataway, NJ).

Total scleral protein was suspended in Laemmli sample buffer, denatured at 95 °C for 5 min and then chilled. Samples of treated, control, and age-matched normal sclera were run in triplicate (typically 8 – 14 µg depending on target protein expression level) on 10% SDS–PAGE resolving gels with a 5% stacking gel, alongside ECL Plex Fluorescent Rainbow markers (GE Healthcare). Gels were electro-blotted onto low-fluorescence PVDF membranes according to standard methods. Blots were blocked for 1 h at room temperature with 5% BSA in tris-buffered saline supplemented with Tween-20 (20 mM tris base, 150 mM NaCl, 0.1% Tween-20, pH 7.6; TBST). The exception was TGFBI which used 5% donkey serum in TBST due to interaction with the secondary antibody by BSA. Blots were probed with human specific primary antibodies (Table 2), multiplexed with the anti-GAPDH antibody, in blocking solution at 4 °C overnight and then washed 3 times with TBST before being probed with Cy3- or Cy5-conjugated secondary antibodies in TBST (1 in 2,500 dilution) at room temperature for 1 h. Blots were again washed 3 times with TBST and then dried before imaging at 100 µm resolution on a Typhoon Trio plus (GE Healthcare) using settings appropriate for the Cy3 and Cy5 fluorophores.

**Table 2:** Human primary antibodies used in the protein expression study.

Protein	Concentration	Host and type	Source
THBS1	0.6 μg/ml	Mouse monoclonal	Gift of Dr. Murphy-Ullrich (UAB); mAb 133 [37]
TGFBI	0.2 μg/ml	Goat polyclonal	R&D Systems; AF3925
TGFB2	0.8 μg/ml	Rabbit polyclonal	Santa Cruz Biotechnology; sc-90
TGFBR3	0.4 μg/ml	Rabbit polyclonal	Santa Cruz Biotechnology; sc-28975
MMP14	0.2 μg/ml	Rabbit polyclonal	Santa Cruz Biotechnology; sc-30074
TIMP3	0.4 μg/ml	Rabbit polyclonal	Santa Cruz Biotechnology; sc-30075
GAPDH	0.4 μg/ml	Rabbit polyclonal	Santa Cruz Biotechnology; sc-25778
GAPDH	0.4 μg/ml	Mouse monoclonal	Santa Cruz Biotechnology; sc-47724

Antibody specificity in tree shrew was validated by antigen blocking or immunoprecipitation followed by mass spectrometry (data not shown).

Blot images were assessed by ImageQuant TL software (GE Healthcare) to automatically detect protein bands and subtract background noise. Protein levels were quantified from the integrated band volume; if multiple protein bands were detected then the sum of the band volumes was used. Relative protein levels were calculated by first normalizing the target protein to the GAPDH reference, and then comparing between treated and control eyes, treated and normal eyes, control and normal eyes. The geometric group mean (for the 5 biological replicates) of these expression ratios was used to calculate the relative fold change in protein expression for each of the target proteins. Paired *t*-tests were used to assess treated versus control eye differences; unpaired *t*-tests were used to test for differences between all independent groups; a p<0.05 was considered significant.

## Results

### Gene expression study

#### Refraction

**Figure 1:**
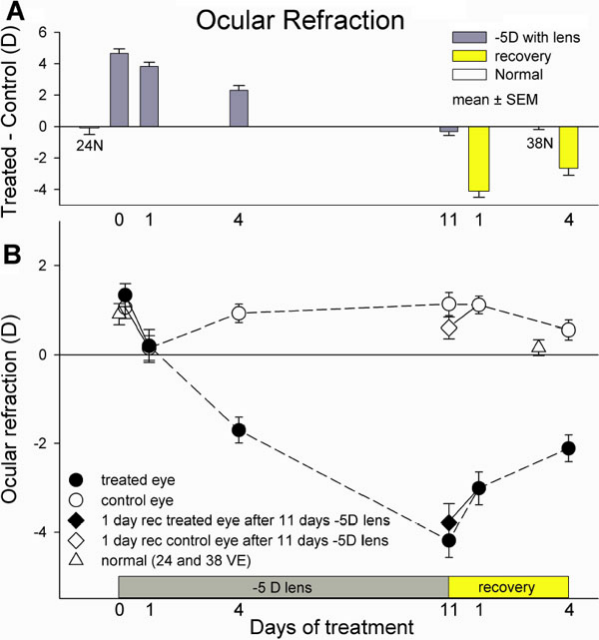
Refractive changes during minus lens treatment and recovery. **A**: Refractive differences (treated eye – control eye). Gray bars (lens-compensation groups) indicate the refractive difference, measured with the –5D lens in place. Yellow bars indicate the amount of myopia after 1 and 4 days of recovery. Normal eyes showed little difference between the right and left eyes. **B**: Refractive values, measured with the –5D lens removed, for the treated eyes (filled circles), control eyes (open circles) and normal eyes (open triangles). Values are corrected for the 4 D small eye artifact [34]. Note that the filled and open circles on day 0 (start of treatment) and day 1 are measurements from the same group of animals. The diamond symbols at 11 days of lens wear, connected with a solid line, are the refractions of the treated and control eyes of the 1 day recovery group measured at the end of lens wear. These are plotted to show the yoking of the control eyes with the treated eyes at the start of lens wear and at the start of recovery. Error bars indicate standard error of the mean (SEM).

The refractive changes produced by –5D lens treatment and recovery are comparable, but not identical, to those observed in previous studies in which tree shrews received similar visual treatments [17,30]. Minus lens treatment initially made the treated eyes hyperopic with the lens in place, relative to the control eyes (Figure 1A) and then produced a rapid compensation by the treated eye so that the with-the-lens hyperopia decreased and the refraction, measured without the lens (Figure 1B) became myopic. After 11 days of treatment, the treated eyes had fully compensated for the –5D lens and were myopic with the lens removed. When lens wear was discontinued, rapid refractive recovery from the induced myopia occurred, with approximately 50% recovery after 4 days. The control eyes of the groups generally showed age-normal refractions. However, there was evidence of a yoked myopic shift in refraction in both the treated and control eyes of the group that had 1 day of lens treatment (Figure 1B). Also, both the treated and control eyes of the group given 1 day of recovery showed a hyperopic shift in refraction. Although A-scan ultrasound was not performed at the end of treatment to avoid any possible effect of anesthesia on gene expression, previous studies have consistently shown that refractive changes produced by minus lens treatment and recovery in tree shrews are due almost entirely to changes in vitreous chamber depth [16,17].

The expression level of mRNA for the reference gene, *POLR2A*, did not vary significantly as a function of age or treatment condition. A 1-way ANOVA comparing the *POLR2A* Ct vales of right, left, treated, and control eyes from all groups did produce a p value of 0.0498. However, Tukey HSD post hoc tests did not identify a significant difference between any two groups. Therefore, the expression of the target genes relative to the expression of *POLR2A* provides a valid comparison between the treated and control eyes and also the eyes of the age-matched normal groups of animals.

#### Differential effects – general patterns

The pattern of differential (treated versus control) changes in scleral mRNA expression levels as a function of time wearing the –5D lens or recovering from lens-induced myopia is consistent with a rapid initiation of scleral remodeling in response to minus lens wear that dissipates as the eyes complete their compensation for the induced refractive error [16,17]. There is an even more rapid change at the onset of recovery. The pattern is also consistent with the previously described modulation of scleral creep rate that occurs during minus lens compensation and recovery [11]. This section will describe the overall pattern of differential gene expression. The following section will examine the specific genes and pathways that showed differential expression.

**Figure 2:**
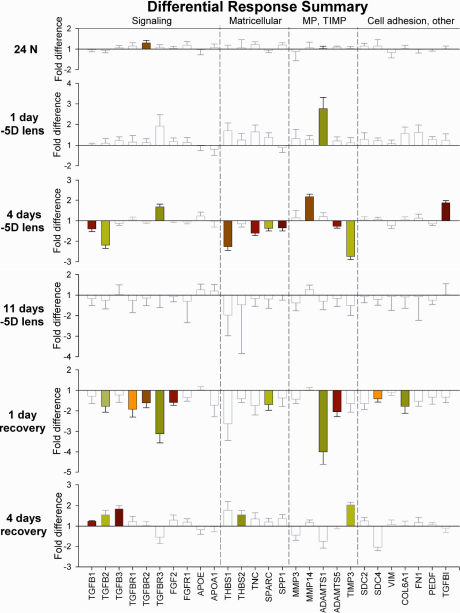
Summary of differential scleral mRNA expression levels in normal animals, in groups after 1, 4 and 11 days of –5D lens wear and after 1 and 4 days of recovery from –5D lens wear. Filled bars indicate that mRNA expression was significantly higher or lower in the treated eye than in the control eye (paired *t*-test, p<0.05). Unfilled, gray bars indicate differences that did not reach statistical significance. Error bars indicate standard error of the mean (SEM).

The mRNA levels in the treated eyes, relative to their fellow untreated control eyes, are summarized in Figure 2 and the left side of Table 3. At some point during lens treatment or recovery a significant difference in mRNA expression was observed in the treated eyes versus the control eyes for 19 of the 27 genes studied.

**Table 3:** Significant mRNA level (fold) differences.

** Gene**	**Treated versus control**					**Treated or control versus normal**										** **
** **	**Lens wear**			**Recovery**		**Lens wear**						**Recovery**				** **
** **	**1 day**	**4 days**	**11 days**	**1 day**	**4 days**	**1 day**		**4 days**		**11 days**		**1 day**		**4 days**		** **
** **	** **	** **	** **	** **	** **	**Tvs24N**	**Cvs24N**	**Tvs24N**	**Cvs24N**	**Tvs38N**	**Cvs38N**	**Tvs38N**	**Cvs38N**	**Tvs38N**	**Cvs38N**	**24N versus 38N**
**Signaling pathways**																
*TGFB1*	–	−1.4	–	–	1.2	−1.3	−1.4	–	–	–	1.5	–	–	1.4	–	–
*TGFB2*	–	−2.2	–	−1.8	1.6	−3.7	−4.2	–	–	–	–	–	–	3.3	2.1	−2.4
*TGFB3*	–	–	–	–	1.8	−2.1	−2.6	–	–	2.1	2.1	–	1.6	2.5	–	–
*TGFBR1*	–	–	–	−1.9	–	–	−2.0	–	–	–	–	−1.8	–	2.0	–	–
*TGFBR2*	–	–	–	−1.6	–	–	−1.5	–	–	–	–	–	–	1.5	1.5	–
*TGFBR3*	–	1.7	–	−3.1	–	–	−3.3	–	–	2.0	2.1	−2.9	–	–	2.1	–
*FGF2*	–	–	–	−1.6	–	−1.8	−2.1	–	–	–	–	−1.8	–	1.6	–	–
*FGFR1*	–	–	–	–	–	−1.8	−2.0	–	–	–	–	–	–	1.7	–	–
*APOE*	–	–	–	–	–	–	–	–	–	–	–	–	–	−2.0	−1.7	–
*APOA1*	–	–	–	–	–	–	–	–	–	–	–	–	–	–	–	–
**Matricellular proteins**																
*THBS1*	–	−2.3	–	–	–	−2.9	−4.8	−3.2	–	–	2.6	–	–	2.4	–	–
*THBS2*	–	–	–	–	1.6	–	−2.4	–	–	–	–	−2.4	–	2.1	–	–
*TNC*	–	−1.6	–	–	–	–	–	–	–	–	–	–	–	–	–	–
*SPARC*	–	−1.4	–	−1.7	–	−2.3	−3.1	−1.7	–	1.8	2.3	–	–	–	–	−1.6
*SPP1*	–	−1.4	–	–	–	–	–	–	–	–	–	–	–	1.6	–	–
**MPs/TIMPs**																
*MMP3*	–	–	–	–	–	–	–	–	–	–	–	–	–	–	2.5	–
*MMP14*	–	2.2	–	–	–	–	–	3.2	–	−4.8	−5.9	−5.9	−5.9	–	–	–
*ADAMTS1*	2.8	–	–	−4.0	–	–	−4.2	–	–	2.0	2.7	−6.3	–	−1.6	–	–
*ADAMTS5*	–	−1.3	–	−2.0	–	−2.7	−3.2	–	–	–	–	−2.4	–	2.1	2.1	−2.0
*TIMP3*	–	−2.8	–	–	2.0	−2.7	−3.0	−2.7	–	–	–	–	–	2.6	–	–
**Cell adhesion/other**																
*SDC2*	–	–	–	–	–	–	−2.1	–	–	–	–	–	–	1.7	–	–
*SDC4*	–	–	–	−1.4	–	–	–	–	–	–	–	–	–	–	–	–
*VIM*	–	–	–	–	–	–	–	–	–	–	–	–	–	−2.2	−2.4	–
*COL6A1*	–	–	–	−1.8	–	−2.2	−3.4	–	–	1.6	1.8	−2.0	–	–	–	–
*FN1*	–	–	–	–	–	−2.5	−4.0	–	–	2.0	2.1	–	–	2.2	–	−2.0
*PEDF*	–	–	–	–	–	−1.9	−2.5	−1.7	−1.4	–	1.7	–	–	–	–	–
*TGFBI*	–	1.9	–	–	–	−1.7	−2.1	–	–	–	–	−3.1	–	–	–	–

Tvs24N indicates comparisons of treated eyes with those of normal animals with 24 days of visual experience (VE); Tvs38N indicates comparison with normal animals with 38 days of VE.

**Figure 3:**
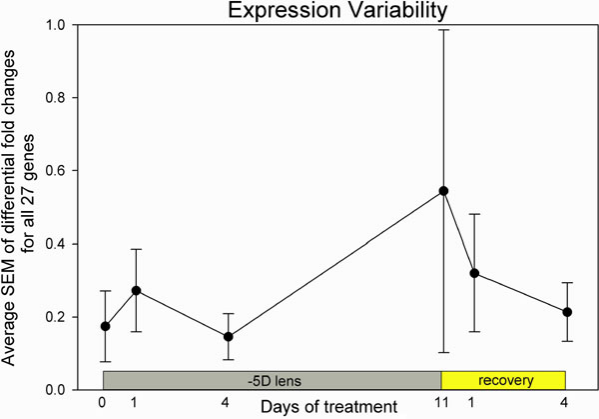
The average of the SEM values of the differential gene expression for all 27 genes is shown for the 24 VE normal group (day 0) and for each of the lens-wear and recovery groups. The error bars indicate the variability (standard deviation) of the SEM values for each group.

To examine the differential effects of monocular minus lens treatment, it is useful to know what the differential (right eye versus left eye) mRNA levels are in age-matched normal eyes (24N in Figure 2). At 24 days of VE, the age when –5D lens treatment was begun, the average difference between the mRNA levels in the right and left eyes of normal animals was very small, and the variability, as measured by the average of the standard error of the mean values for the right-eye versus left-eye expression levels of the 27 genes was very low (0.17±0.10 fold, mean±SD; Figure 3). Expression for eight genes was slightly lower in the right eye and expression for 19 was slightly higher. This pattern of up versus down differences was not significantly different from what would be expected from random variability (sign test, p>0.05). Expression for one gene (*TGFBR2*) was significantly higher (paired *t*-test, p<0.05), primarily because the variability of the mRNA level differences across the eight animals in the group was extremely low.

After one day of –5D lens wear, three changes were noted: 1) mRNA expression for one gene (*ADAMTS1*) was significantly upregulated in the treated eyes (Figure 2, paired *t*-test, p<0.05); 2) the variability in gene expression between the treated and control eyes was elevated (0.27±0.11 fold; Figure 3); and 3) as shown by the gray outlines in Figure 2, the treated eye mRNA expression for 23 of 26 genes was higher, but not significantly, than expression in the control eyes. If one were to assume that normally the differential expression would randomly vary between eyes, with some expression levels higher in one eye and the rest higher in the other eye, as in the normal animals at 24 days of VE, the bias after one day of treatment for the treated-eye mRNA expression levels to be higher than the control-eye values may be an early sign of differential changes. This upward bias in the direction of the non-significant changes in expression level was significantly different from random (sign test, p<0.05).

After 4 days of –5D lens wear (Figure 2), when the treated eyes were rapidly compensating for the lens, 11 genes showed significant differential expression (p<0.05, paired *t*-test) as described in the next section. The direction of the non-significant differential expression levels was evenly divided; expression was higher in 8 and lower in 8 genes. Variability of the differential expression levels was low (0.15±0.06 fold), as reflected in the smaller SEM values (Figure 3). The mRNA expression levels from this group were also compared with normal eyes measured at 24 days of VE as shown on the right side of Table 3 (“lens wear, 4 days”). The control eye mRNA levels were generally not significantly different from the normal animals, while mRNA levels for five genes were lower than normal in the treated eyes.

After 11 days of –5D lens wear, when the treated eyes had compensated for the lens (all were within 1.2 D of full compensation) there were no significant differences in mRNA expression between the treated and control eyes (paired *t*-test, p>0.05); overall, the pattern was for lower expression in the treated eyes (lower than control eye for 22 of 27 genes; sign test, p<0.05). Differential expression in this group was characterized by very high variability (0.54±0.44 fold; Figure 3). This was significantly greater variability than found in any of the other groups (1-way ANOVA across all groups, p<0.00000; Tukey HSD post hoc test, p<0.05, 11 days −5D versus each other group). The mRNA expression levels from this group were also compared with normal eyes measured at 38 days of VE as shown on the right side of Table 3 (“lens wear, 11 days”). For the genes that showed a significant difference, the pattern was primarily upregulation for both the treated and control eyes.

After 1 day of recovery (Figure 2), mRNA expression of 25 genes was lower in the recovering eye compared to the control eyes. This was significantly different from a random distribution of up- and downregulation (sign test, p<0.05). For 10 genes the expression was significantly lower (Figure 2, paired *t*-test, p<0.05). The variability across animals within the group (0.32±0.16 fold) was significantly higher in comparison to that of the group with 4 days of lens wear (Figure 3), but the variability was smaller than in the group after 11 days of compensation. Comparing the pattern of differential expression after one day of lens wear with one day of recovery (Figure 2), there were more significant differential changes after one day of recovery than after one day of –5D lens treatment (filled bars in Figure 2).

After 4 days of recovery, mRNA levels for 5 genes were significantly upregulated (paired *t*-test, p<0.05). Levels for 8 other genes were non-significantly higher and 8 were lower, also not significantly. Variability of the differential gene expression levels was relatively low (0.21±0.08 fold, Figure 3) and not significantly different from the variability of the normal animals measured at 24 days of visual experience.

#### Differential effects – pathways and specific genes

The statistically significant differential changes in gene expression found in this study (filled bars in Figure 2) expand upon the suggestion from previous studies [16,30] that retinally-derived “go” and “stop” signals produce a complex, selective remodeling of the scleral extracellular matrix. Differential changes in mRNA levels for signaling proteins, matricellular proteins, MPs, and TIMPs, along with cell adhesion and other proteins were consistent with a pattern, during lens compensation, of reduced ECM synthesis, increased ECM degradation, and reduced matricellular, proteoglycan core proteins, and cell adhesion protein synthesis. The pattern of differential mRNA changes during recovery suggested a partial reversal of the pattern during lens compensation; four genes (*TGFB2*, *TGFBR3*, *ADAMTS1*, and *TIMP3*) showed differential mRNA regulation in opposite directions during minus lens wear and recovery. However, other mRNAs that did not differentially change during lens compensation were differentially expressed during recovery (Figure 2, Table 3).

Differential changes were found in the mRNA levels of genes in the *TGFβ* signaling pathway. After 4 days of minus lens wear, two *TGFβ* isoforms (*TGFB1*, *TGFB2*) were downregulated (–1.4 and –2.2 fold) and one *TGFβ* receptor (*TGFBR3*) was upregulated (+1.7 fold). After 1 day of recovery, *TGFB2* and all three *TGFβ* receptors were downregulated (–1.8, –1.9, –1.6, –3.1 fold). After 4 days of recovery, mRNA levels for all three *TGFβ* isoforms were upregulated (+1.2, +1.6, +1.8 fold) while the mRNA levels for the three *TGFβ* receptors were not significantly different. In general, mRNA levels for *TGFβ* and its receptors were regulated in opposite directions. The changes in the *TGFβ* signaling pathway are consistent with previous studies [31] that suggest *TGFβ* is involved in scleral remodeling during lens compensation and recovery. The only effect on the FGF signaling pathway was downregulation of *FGF2* mRNA after 1 day of recovery. Neither *APOA1*, which has been implicated in chick sclera [20], nor *APOE*, which often serves a role similar to *APOA1* in mammals [37,38], showed statistically significant differential changes during lens wear or recovery.

Differential changes were observed in the mRNA levels of all five matricellular proteins examined. After 4 days of –5D lens wear, *THBS1*, *TNC*, *SPARC*, and *SPP1* were all downregulated (–2.3, –1.6, –1.4, –1.4 fold). After 1 day of recovery *SPARC* was downregulated (–1.7 fold) and after 4 days of recovery *THBS2* was upregulated (+1.6 fold).

Consistent with previous studies [16], there were differential effects on *MP* and *TIMP* mRNA levels suggesting increased degradation during lens compensation and a partial reversal during recovery. mRNA for *ADAMTS1*, an aggrecanase, was upregulated (+2.8 fold) after 1 day of minus lens wear. After 4 days of lens wear, *MMP14* mRNA was upregulated (+2.8 fold) in treated eyes while *ADAMTS5* and *TIMP3* mRNA levels were downregulated (–1.3, –2.8 fold). After 1 day of recovery, mRNA levels for *ADAMTS1* and *ADAMTS5* were both downregulated (–4.0, –2.0 fold). After 4 days of recovery *TIMP3* mRNA was upregulated (+2.0 fold). *ADAMTS1* and *TIMP3* were two of four genes that were differentially regulated in opposite directions during lens compensation and recovery. Gene expression for *MMP3* was not differentially regulated.

mRNA levels for *TGFBI*, which has been implicated in decreasing the adhesion of fibroblasts to collagen matrix [39] was upregulated at 4 days of lens wear (+1.9 fold). *SDC4* and *COL6A1* mRNA levels were downregulated after 1 day of recovery (–1.4, –1.8 fold).

#### Binocular changes

**Figure 4:**
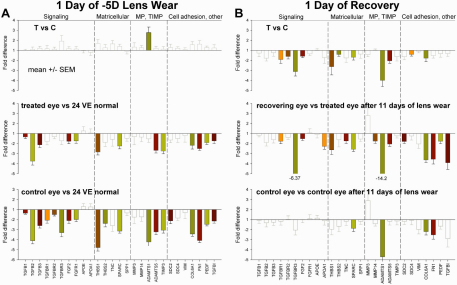
Summary of the early scleral mRNA responses. **A**: Differences after one day of lens wear. Top row: Fold difference between the treated and control eyes after 1 day of –5D lens wear, as shown in Figure 2. Middle row: Treated eye scleral mRNA levels relative to those of eyes in the 24 VE normal group. Bottom panel: Control eye scleral mRNA levels relative to those of eyes in the 24 VE normal group. **B**: Differences after one day of recovery. Top row: Fold difference between the treated and control eyes after 1 day of recovery, as shown in Figure 2. Middle row: Treated eye scleral mRNA levels relative to treated eyes in the group with 11 days of –5D lens treatment. Bottom panel: Control eye scleral mRNA levels relative to those of control eyes in the group with11 days of –5D lens treatment. Filled bars indicate that the difference was statistically significant. Paired *t*-test, p<0.05, for treated control eye comparisons (top panels); unpaired *t*-test for other comparisons (middle and bottom panels). Unfilled, gray bars indicate differences that did not reach statistical significance. Error bars indicate standard error of the mean (SEM).

In addition to examining differential expression levels between the treated and control eyes, we also compared the mRNA expression levels in the treated and control eyes with expression levels in normal animals. The top panel of Figure 4A repeats the 1 day differential results from Figure 2. The middle and bottom panels and the right side of Table 3 (“lens wear, 1day”) show the mRNA levels in the group with 1 day of –5D lens wear compared with the normal group with 24 days of VE. In this comparison, we assumed that the mRNA expression values for the normal group (average of right and left eyes) were the baseline against which the 1day of lens wear group should be compared. The changes from the normal animals indicate the initial response of both the control and treated eyes to monocular hyperopia. The middle panel shows the difference in mRNA expression between the treated eyes versus normal eyes; the bottom panel shows the control eye versus normal eye difference. The dominant early response to hyperopia (–5D lens) was downregulation in both the treated and control eyes (sign test p<0.05). Nineteen of the 27 genes showed significant downregulation (unpaired *t*-test, p<0.05). In both eyes, mRNA expression levels for 13 genes were significantly lower. In addition, expression levels for another 6 genes were significantly lower in the control eyes compared to normal. This yoked response contrasted with the differential effects (top panel), where a significant difference was found in the mRNA levels for only one gene (*ADAMTS1*), and the direction was upregulation of the treated eyes. The overall pattern of the treated eye versus control eye differences was upregulation of mRNA levels (top panel of Figure 4A), but, relative to normal, the response in the treated eyes was downregulation. The differential trend toward upregulation occurred because the mRNA of the control eyes was downregulated, relative to normal, by a larger amount than was the case for the treated eyes.

We also compared the mRNA expression levels of the control and treated eyes of the animals with one day of recovery with two groups. One comparison was with the group with that wore the lens for 11 days and had compensated for the –5D lens (Figure 4B). In this comparison, we assumed that the mRNA expression values for the treated and control eyes of the 11 day lens-wear group were the baseline against which the 1 day of recovery group should be compared; both groups wore the lens for the same time period and compensated similarly (Figure 1B), but the 1 day recovery group was measured a day later, after 24 h with the lens removed. The changes of both the control and treated eyes from this group indicate the initial response to monocular myopia. Interestingly, the dominant early response to myopia (recovery), like the response to hyperopia (Figure 4A), was mRNA downregulation in both the treated and control eyes (sign test p<0.05). The differential downregulation at 1 day of recovery (top panel in Figure 4B) was due to a larger downregulation in the treated eyes than in the control eyes.

The other comparison of the recovery groups was with the normal animals at 38 days of VE. The pattern for the animals with 1 day of recovery was similar to that described above, a general downregulation of the treated eyes and, to some extent, the control eyes. The group with four days of recovery showed numerous significant differences (p<0.05, unpaired *t*-test) between the treated and control eye mRNA levels and normal levels, including both up- and downregulation in the treated and control eyes compared to normal values.

#### Overall parallel patterns of mRNA levels

**Figure 5:**
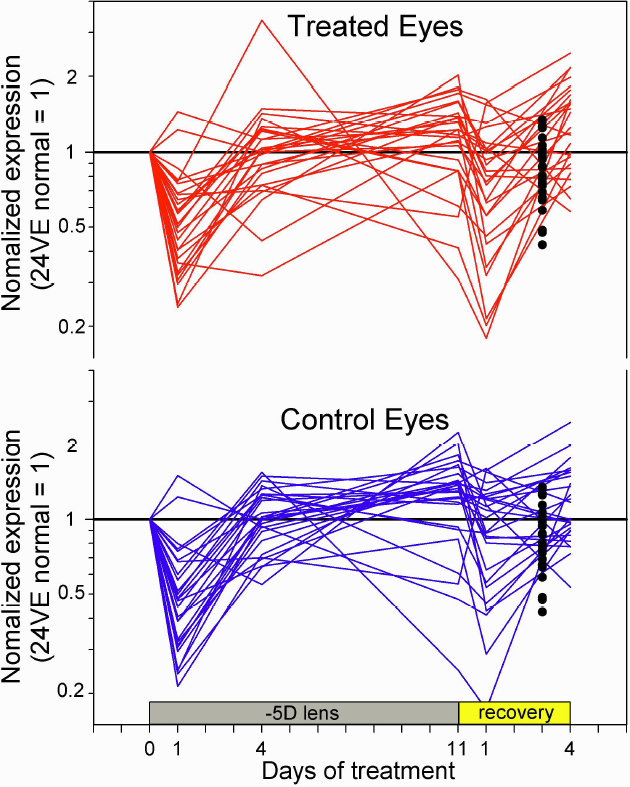
Expression of all genes in the treated and control eyes normalized to the levels of the normal animals measured at 24 days of VE. The filled circles at 3 days of recovery are the 38 VE normal mRNA values normalized to the 24 day normal values.

In addition to the initial yoked downregulation of mRNA levels in the treated and control eyes, there is an overall parallel pattern of the control and treated eyes across all of the lens wear and recovery groups. Figure 5 shows the mRNA expression levels in the treated and control eyes at each time point of –5D lens wear and recovery relative to the initial values measured at 24 days of VE. It also compares the older (38 days of VE) normal group with the 24 day normal group, and shows that there was a small downward trend in mRNA levels as a function of age. Superimposed on this is a pattern that is similar for most of the genes and for the treated and the control eyes in the treated groups. The relative downregulation in the treated and control eyes at 1 day of –5D lens and again after 1 day of recovery, described in Figure 4, can clearly be seen here. Beyond that, however, the treated and control eye mRNA levels trended in the same direction, up toward the original normal levels after 4 and 11 days of lens wear and, for both eyes, back toward the original normal levels after 4 days of recovery. Thus, there appeared to be an overall pattern of yoking of the treated and control eyes throughout lens compensation and recovery.

### Protein expression study

#### Refraction

The refractive changes produced by –5D lens treatment and recovery in the western blot animals (data not shown) were comparable to the PCR animals and previous studies [17,30].

#### Western blotting

Six proteins (TGFB2, TGFBR3, THBS1, TGFBI, MMP14, and TIMP3) that showed differential mRNA expression after 4 days of –5D lens wear were examined by western blotting. GAPDH was measured as the reference protein. After 4 days of –5D lens wear, the abundance of two proteins was significantly lower in the treated eyes relative to the control eyes: TGFBR3 (–1.2 fold) and THBS1 (–4.9 fold). After 4 days of recovery, none of the proteins examined showed a significant difference in abundance between the treated and the control eyes. There were no significant differences between right and left eye protein levels or between levels in the young (24 days of VE) versus older animals (39 days of VE).

#### Comparison with mRNA results

Overall, there was not good agreement between the mRNA and protein data. Table 4 compares the differential mRNA expression patterns with the differential protein results for the two treatment conditions examined (4 days of lens wear and 4 days of recovery). The treated versus control eye fold difference for the proteins is shown followed by NS if the difference did not reach statistical significance (p>0.05, paired *t*-test). In two cases, (THBS1 and TGFBR3 after 4 days of lens wear) both the differential mRNA expression and the differential protein expression reached statistical significance. In one case (THBS1) both increased. In the other (*TGFBR3*) the treated eye mRNA levels were upregulated while the protein levels were downregulated. There were three additional instances where the protein levels changed in the same direction as the mRNA levels (indicated with bolding of the type), but the differential protein expression did not reach statistical significance.

**Table 4:** Differential mRNA and protein comparison.

** Gene**	**4 Days –5D Lens**	**4 Days Recovery**
*TGFB2* mRNA	**−2.2**	1.6
protein	**−1.3 NS**	−1.3 NS
*TGFBR3* mRNA	1.7	−1.5 NS
protein	−1.2	1.0 NS
*THBS1* mRNA	**−2.3**	**1.8 NS**
protein	**−4.9**	**3.2 NS**
*TGFB1* mRNA	1.9	**−1.1 NS**
protein	1.0 NS	**−1.2 NS**
*MMP14* mRNA	2.3	1.2 NS
protein	1.0 NS	1.0 NS
*TIMP3* mRNA	−2.8	2.0
protein	1.0 NS	−1.1 NS

Values are fold changes. Bold indicates changes in the same direction.

## Discussion

The results of this study add to our understanding of the scleral changes that occur during minus lens compensation and recovery by revealing the patterns of gene expression both across a large number of candidate genes, and also as a function of time. Distinctive mRNA expression patterns were found: both differential patterns and yoked, directional patterns that changed over time for genes in four categories 1) signaling pathways, 2) matricellular proteins, 3) *MP*s and *TIMP*s, 4) cell adhesion and other proteins. These patterns suggest that genes within each group may participate at differing times to produce remodeling of the sclera that results in accelerated axial elongation in response to hyperopia and reduced axial elongation in response to myopia.

### Differential patterns

Differential patterns, in which the treated eyes change from the control eyes, are of interest because it is the treated eyes that increase their creep rate and axial elongation rate during lens compensation and decrease them during recovery, whereas the control eyes remain relatively, but not completely, normal [11].

#### Signaling pathways

The changes in the mRNA levels of the *TGFβ* isoforms (decreased at 4 days of lens wear, increased at 4 days of recovery) confirm and extend findings from previous studies using form deprivation that suggested the involvement of the *TGFβ* signaling pathway [31]. A novel finding of this study is the modulation of mRNA levels for all three *TGFβ* receptors. This suggests that receptor expression, in addition to expression of the *TGFβ* isoforms, may be involved in *TGFβ* signaling in tree shrew sclera during lens compensation and recovery. Of particular interest is the increased mRNA expression after 4 days of lens wear, and decreased expression after 1 day of recovery, of the non-signaling receptor *TGBFR3*, also known as betaglycan. Betaglycan regulates TGFβ access to the signaling receptors. When anchored in the cell membrane, betaglycan facilitates signaling by presenting TGFβ to the signaling TGFβ receptors [40]. In contrast, soluble betaglycan is a potent inhibitor that sequesters TGFβ in the extracellular space preventing it from binding to the signaling receptors [41]. Soluble betaglycan is produced from the membrane-anchored form by proteolytic cleavage, a process that is thought to be mediated by MMP14 [42]. The relative increase in mRNA at 4 days for both betaglycan and *MMP14* is consistent with the idea that there may be increased production of the inhibitory, soluble betaglycan. If there were more soluble betaglycan and less TGFβ, as suggested by the decrease in *TGFβ* mRNA, there could be an overall reduction in TGFβ signaling during lens compensation. During recovery there was an increase in *TGFβ* mRNA, a decrease in betaglycan mRNA, and *MMP14* mRNA returned to normal levels in the treated eyes, which might combine to increase TGFβ pathway signaling.

In chicks, apolipoprotein–A1 has been suggested to be a “stop” signal in retina and sclera [20]. However, mRNA levels for *APOA1* and apolipoprotein-E, which in mammals plays a similar role to *APOA1* [37.38], did not show differential changes during lens compensation or recovery. However, mRNA levels for a gene from the fibroblast growth factor signaling pathway, *FGF2*, were downregulated after 4 days of recovery.

#### Matricellular proteins

Matricellular proteins are non-enzymatic, non-structural proteins that are thought to modulate interactions between various ECM components [43]. This study found differential changes in the mRNA levels of all of the candidate genes (*SPARC*, *THBS1*, *THBS2*, *TNC*, *SPP1*) suggesting general involvement of the matricellular proteins in scleral tissue remodeling during lens compensation and recovery. The significant differential downregulation of 4 of the 5 after 4 days of –5D lens wear when the increase in scleral creep rate and increase in axial elongation rate are at a maximum [11] suggests that a reduction in matricellular protein-related activity may play a role in increasing scleral creep rate.

#### MPs and TIMPs

The findings of this study support previous studies that suggest the involvement of MPs and TIMPs in scleral tissue remodeling [16,44-46]. The levels of MMP14, a membrane bound MMP that activates proMMP-2, directly cleaves collagen and proteoglycan core proteins, and produces soluble betaglycan, were higher in the treated eye sclera after 4 days of –5D lens treatment while the levels of TIMP3, an inhibitor of MMP14, were lower. Together, these changes in gene expression could potentially increase the activity of MMP14 which in turn may contribute to the increase in scleral creep rate by degrading collagen fibrils at the edges of the scleral lamellae, increasing the ease with which the lamellae slip across each other.

Two metalloproteinases, not previously studied in tree shrew, ADAMTS1 and ADAMTS5, also known as aggrecanase 3 and aggrecanase 2, respectively, were considered good candidates for examination because of their potential to cleave the core protein of the large proteoglycan aggrecan. Previous studies have shown that the fibrous sclera, surprisingly, contains relatively large amounts of this cartilage proteoglycan, and that scleral aggrecan mRNA levels are decreased during lens compensation and increased during recovery in tree shrews [30,47]. The modulation of ADAMTS1 appeared as an early, transient response to both hyperopia and myopia. After 1 day of –5D lens treatment the mRNA levels for *ADAMTS1* were nearly threefold higher in the treated eye sclera than in the control eye sclera and after 1 day of recovery they were fourfold lower. Both initial changes were largely gone at 4 days of lens treatment and 4 days of recovery. These data suggest that an increase (lens wear) and decrease (recovery) in aggrecan cleavage coupled with a decrease (lens wear) and increase (recovery) of aggrecan mRNA, might be early steps in the tissue remodeling processes that quickly changes aggrecan levels. Changes in aggrecan content, which, due to its large size is located between the scleral lamella [30], may contribute to the changes in GAG content [17], and the modulation of scleral creep rate during lens compensation and recovery.

#### Cell adhesion and other proteins

Only three of the seven candidate genes examined in this category showed differential changes. mRNA for *TGFBI*, which has been implicated in decreasing the adhesion of fibroblasts to collagen matrix [39], was increased at 4 days of lens wear while mRNA for *SDC4* and *COL6A1* were reduced after 4 days of recovery. mRNA for vimentin (*VIM*), an intermediate filament protein involved in cytoskeletal changes, that has been found to upregulate in response to form deprivation in chick retina [20], did not show differential changes at any point in lens compensation or recovery.

### Global patterns of differential changes

In addition to the individual differential changes that reached statistical significance, general patterns of differential changes emerged that appeared to reflect overall scleral responses to lens compensation and recovery. As noted in Figure 2, the difference in mRNA expression, across all genes, between the right and left eyes of normal animals was very small, and the variability was very low. This pattern was significantly altered throughout the 11 days of –5D lens wear and 4 days of recovery. Thus, larger differences and/or higher variability provide a sense of the dynamics of scleral gene expression and appear to be general indicators that the scleral fibroblasts were responding to the retinally-derived signals produced by hyperopia and myopia.

There was an overall trend toward higher expression levels in the treated eyes relative to control eyes (Figure 2) after 1 day of lens wear that transitioned to selective bidirectional modulation after 4 days that suggests that there is selective regulation of the mRNA levels during the most rapid axial elongation phase with the highest scleral creep rate. After 11 days of lens treatment, when most eyes had fully compensated for the lens and (as shown in prior studies [11]) axial elongation rate had slowed, there were no significant mRNA differences. However, the variability between treated and control eye mRNA levels was very high, suggesting the scleras had not returned to a normal state. This is consistent with the fact that the sclera is in an elongated state, relative to normal, and that the scleral creep rate is still above normal levels [48].

The high variability in mRNA levels present after 11 days of lens compensation (Figure 3) rapidly transitioned, after 1 day of recovery, to overall lower variability and significantly lower mRNA levels in the treated eyes versus the control eyes (Figure 2). This suggests that there is a rapid, strong differential response to the initial myopia that is opposite in direction to the overall upward differential response to the initial hyperopia. The rapid (1 day) emergence of significantly downregulated mRNA levels in the recovering eyes, in contrast to the slower emergence of significant differential mRNA levels at the onset of lens wear (compare 1 day lens wear versus 1 day recovery in Figure 2) may suggest a stronger response to myopia than to hyperopia, or may reflect the fact that the sclera, after 11 days of lens compensation, is not normal and may to be able to respond quickly to the initial myopia at the onset of recovery. Indeed, measures of scleral creep rate after 1 day of recovery have found that scleral creep rate drops rapidly (Siegwart, unpublished data, 2007). This rapid transition may also provide insight into the difference between the response of the eyes during recovery (when their elongation and creep rates drop below normal) versus the response of normal age-matched animals that begin to wear plus lenses and show little change in the axial elongation rate in response to a similar amount of myopia [10]. After 4 days of recovery, the pattern of mRNA differential expression had transitioned to a bidirectional pattern that was similar to the transition from 1 to 4 days of –5D lens treatment.

### Global binocular changes

As shown in Figure. 4, Figure 5, and in Table 3, the dominant early scleral mRNA response to both the onset of monocular hyperopia (1 day of –5D lens wear) and myopia (1 day of recovery) was lower mRNA levels in both the treated and control eyes. Two aspects of this finding are of interest.

First, the untreated control eye scleras responded, and followed the same initial pattern as the treated eye scleras. It is not clear how a monocular treatment produces changes in the control eyes, but control-eye yoking and anti-yoking has been reported in several different species [11,16,49-52]. It is possible, but seems unlikely, that wearing the –5D lens produced changes in the behavior of the animals (head position, pupillary constriction, etc.) that altered the visual experience of the control eyes. It seems more likely that activity levels in centrally-mediated binocular efferent pathways are altered. For instance, in monocularly treated chicks, there is a binocular decrease in choroidal blood flow that is greater in the treated eye, but nonetheless produces a substantial reduction in choroidal blood flow in the control eyes [53]. Such a change, if it occurred in tree shrews, could affect the global metabolism of the control eyes.

Second, the treated-eye scleras initially responded the same way to both hyperopia and myopia. This finding raises the possibility that the initial scleral response to hyperopia and myopia is similar, possibly a general tissue remodeling response, that is then fine-tuned to produce the changes that lead to opposite changes in scleral creep rate.

The yoked downregulation in both the treated and control eyes, which is not apparent in the differential data that showed a trend toward higher levels in the treated eyes after 1 day of –5D lens and lower levels after 1 day of recovery, clouds the issue of how one should interpret a difference between the treated and control eye. What does it mean, functionally, if there is differential upregulation in the treated eye but the actual overall mRNA levels in that treated eye are decreased from normal? Generally, a treated versus control eye difference is interpreted as a change in the treated eye because the untreated control eye is considered to be relatively normal. The data from this study suggest that this assumption may be insufficient if the point is to determine how gene expression actually changed in the treated eye and produced changes in the scleral biochemistry. Presumably, both the difference between the treated and control eyes and the actual change in the treated and control eye scleras are important. Possibly, it is the relative levels of expression of different genes and their subsequent interaction that has functional consequences rather than the absolute level of expression of a particular gene.

The pattern of directional changes during lens compensation and recovery where the mRNA levels of most of the genes moved in the same direction from one time point to the next in both the treated and control eyes (Figure 5) suggests there may be a global mechanism that affects the expression of many genes simultaneously during lens compensation and recovery. On this background of global directional change there appear to be superimposed more specific individual changes that create treated versus control eye differences. How global directional changes might be induced is unclear, particularly since they also occur in the untreated control-eye sclera.

### Protein levels versus mRNA levels

Overall, there was poor agreement between the mRNA and protein data, which is not surprising given that many studies have shown poor correlation between mRNA and protein levels under a variety of conditions [54]. Both are snapshots that do not capture the dynamic properties of either transcription or translation into protein. Without knowledge of the specific reason for a lack of correlation between steady-state mRNA and protein levels, a change in steady mRNA level without a corresponding change in the steady-state level of the protein cannot be dismissed as functionally unimportant. In particular, it should be kept in mind that a change in steady-state mRNA levels, regardless of the consequences of the change, may indicate a response by the cell to an external signal which may be important in understanding the signaling pathways involved.

### Conclusions

The results of this study suggest that the regulation of scleral gene expression during lens compensation and recovery is in some respects straightforward, and in other respects very complex. It is clear that the visually-guided emmetropization mechanism rapidly regulates scleral gene expression in a highly controlled fashion. Genes in a variety of pathways are involved and there is evidence that there may be underlying global shifting of gene expression that needs to be taken into account. The yoking of treated and control eye gene expression and the subsequent question of the functional meaning of treated versus control eye differences adds complexity to any interpretation of the changes in gene expression. It does not appear that the regulation of a few key genes in the treated eye sclera will be sufficient to explain the tissue remodeling and changes in the mechanical properties of the sclera. This is not particularly surprising given that tissue remodeling, which has been studied for many years in other tissues, is known to be complex, and there is no particular reason to assume that it would be any less complicated in the sclera.

The results of this study, like most previous studies, suggest that the magnitude of scleral gene expression changes during lens compensation and recovery are small. A potentially important point to be taken from the directional changes, that were highly significant by sign test while many of the individual gene expression changes were not statistically significant, is that numerous small changes in gene expression may combine to produce a larger effect. While many of these small changes may not, individually, reach statistical significance because of small sample size, their combined effect may be the tissue remodeling process that we are attempting to explain. The changes observed in this study suggest that understanding scleral gene expression may require an understanding of patterns of gene expression, not just individual significant differences between the treated and control eye.
